# Long-term follow-up of chronic central serous chorioretinopathy patients after primary treatment of oral eplerenone or half-dose photodynamic therapy and crossover treatment: SPECTRA trial report No. 3

**DOI:** 10.1007/s00417-022-05836-x

**Published:** 2022-10-07

**Authors:** Helena M. A. Feenstra, Elon H. C. van Dijk, Thomas J. van Rijssen, Roula Tsonaka, Roselie M. H. Diederen, Carel B. Hoyng, Reinier O. Schlingemann, Camiel J. F. Boon

**Affiliations:** 1 Department of Ophthalmology, Leiden University Medical Center, Albinusdreef 2, 2333ZA Leiden, The Netherlands; 2 Department of Biomedical Data Sciences, Leiden University Medical Center, Leiden, The Netherlands; 3 Department of Ophthalmology, Amsterdam University Medical Centers, University of Amsterdam, Amsterdam, The Netherlands; 4 Department of Ophthalmology, Radboud University Medical Center, Nijmegen, The Netherlands; 5 Department of Ophthalmology, University of Lausanne, Jules-Gonin Eye Hospital, Fondation Asile des Aveugles, Lausanne, Switzerland

**Keywords:** Central serous chorioretinopathy, Eplerenone, Long-term follow-up, Mineralocorticoid receptor antagonist, Photodynamic therapy, SPECS, SPECTRA trial

## Abstract

**Purpose:**

Comparing anatomic and functional efficacy and safety of primary treatment with either half-dose photodynamic therapy (PDT) or oral eplerenone, or crossover treatment in chronic central serous chorioretinopathy patients.

**Methods:**

After the SPECTRA trial baseline visit, patients were randomized to either half-dose PDT or eplerenone and received crossover treatment if persistent subretinal fluid (SRF) on optical coherence tomography (OCT) was present at first follow-up (at 3 months). Presence of SRF and best-corrected visual acuity (BCVA) was evaluated at 12 months.

**Results:**

Out of the 90 patients evaluated at 12 months, complete SRF resolution was present on OCT in 43/48 (89.6%) of patients who were primarily randomized to half-dose PDT and in 37/42 (88.1%) who were primarily randomized to eplerenone. Out of the 42 patients that were primarily randomized to eplerenone, 35 received crossover treatment with half-dose PDT. The BCVA improved significantly more at 12 months in patients who had received primary half-dose PDT as compared to the primary eplerenone group (p = 0.030).

**Conclusions:**

Twelve months after baseline visit, most patients treated with half-dose PDT (either primary or crossover treatment) still had complete SRF resolution. The long-term BCVA in patients who receive primary half-dose PDT is better than in patients in whom PDT is delayed due to initial eplerenone treatment with persistent SRF.

**Supplementary Information:**

The online version contains supplementary material available at 10.1007/s00417-022-05836-x.


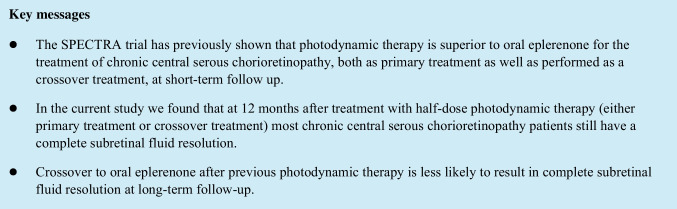


## Introduction

Central serous chorioretinopathy (CSC) is a chorioretinal disease that causes detachment of the neuroretina due to accumulation of subretinal fluid (SRF). This accumulation of SRF, typically under the macula, is thought to be secondary to choroidal abnormalities that induce damage to the outer blood-retina barrier at the level of the retinal pigment epithelium [1–3]. Several risk factors have been proposed for CSC, such as male gender, age of 20–60 years, the use of corticosteroids, and genetic risk factors [4–11]. There are many causes of serous maculopathy, and the distinction from other etiologies from CSC has profound impact on the prognosis and therapeutic decision-making [12].

The classification of CSC is subject of controversy, but CSC can roughly be categorized into acute and chronic (cCSC) forms [4, 13]. Patients with acute CSC are likely to have a spontaneous resolution of SRF within a few months after onset of disease, with minimal accompanying atrophic changes of the retinal pigment epithelium (RPE), whereas cCSC patients have persistent SRF and atrophic RPE, which may lead to permanent damage to the photoreceptors [4, 14, 15]. When the SRF involves the fovea, this may result in a decrease of visual acuity, metamorphopsia, and diminished contrast vision, impacting the quality of life [16–18].

Commonly used treatments for cCSC include half-dose photodynamic therapy (PDT), oral eplerenone, and high-density subthreshold micropulse laser [4]. Several recent randomized clinical trials have been published on the treatment of cCSC. First of all, the PLACE trial and PLACE trial follow-up studies, such as the REPLACE trial, concluded that half-dose PDT is superior to high-density subthreshold micropulse laser treatment in achieving a complete resolution of SRF on optical coherence tomography (OCT) scanning [19–21]. The VICI trial was another large, randomized controlled trial, showing that treatment with oral eplerenone is not superior to placebo in terms of SRF resolution and functional parameters at 1 year after baseline visit [22]. The third large, randomized controlled trial was the SPECTRA trial, in which we compared half-dose PDT to oral eplerenone treatment. At 3 months after treatment, only 17% of patients in the eplerenone group had a complete resolution of SRF on OCT, compared to 78% of patients in the half-dose PDT group. In addition, the mean retinal sensitivity on microperimetry improved significantly more in the half-dose PDT group compared to the eplerenone group [23]. Patients who had SRF at the evaluation visit at 3 months after (the start of) treatment were included in the SPECS trial, receiving crossover treatment, with a subsequent evaluation 3 months later. At 3 months after crossover treatment, 876.5% of cCSC patients in the crossover to half-dose PDT group and 22% in the crossover to eplerenone group had complete SRF resolution, which was a significant difference in favor of half-dose PDT. The mean foveal sensitivity increased significantly more in the patients receiving crossover treatment with half-dose PDT after previous unsuccessful eplerenone treatment, compared to the patients receiving crossover treatment with eplerenone after previous unsuccessful half-dose PDT [24].

Although half-dose PDT appears to be the treatment of choice in cCSC, it is unknown if half-dose PDT is also superior to eplerenone treatment on long-term follow-up. In this follow-up study of the SPECTRA and SPECS trial, we describe the outcome of cCSC patients at 12 months after the primary treatment with either eplerenone or half-dose PDT and crossover treatment, in terms of the desired anatomical effect (complete SRF resolution on OCT) and functional parameters.

## Methods

This prospective multicenter study included patients from the SPECTRA trial (clinicaltrial.gov identifier, NCT03079141). The protocol of this trial has already been published [23, 24]. Patients were included from 3 academic tertiary referral centers located in the Netherlands: Leiden University Medical Center (Leiden), Amsterdam University Medical Centers (Amsterdam), and Radboud University Medical Center (Nijmegen). This study adhered to the tenets of the Declaration of Helsinki and written informed consent was obtained from all participants. Institutional Review Board Committee approval was obtained from all participating centers before the start of the study. Ethics approval was obtained from the medical ethical committee of Leiden University Medical Center (NL59158.058.16).

In the SPECTRA trial, cCSC patients over the age of 18 years with SRF on OCT were randomized (at a 1:1 ratio) to treatment with either oral eplerenone or half-dose PDT (Online Resource 1). At 3 months after (the start of) receiving their assigned treatment, presence of SRF was evaluated on OCT. If SRF was still present at this first evaluation visit (evaluation visit 1), patients were eligible for crossover treatment and were also included in SPECS (central Serous chorioretinopathy treated with half-dose PDT or Eplerenone Crossover Study). In the SPECS trial, the effect of crossover treatment was evaluated at 3 months after attending evaluation visit 1 and receiving crossover treatment (evaluation visit 2). In the current study, patients who could be included in the SPECTRA trial received follow-up at 12 months after baseline visit (evaluation visit 3). The primary outcome measure was the odds ratio for presence of SRF on OCT at this evaluation visit 3. The secondary endpoints included best-corrected visual acuity (BCVA) in Early Treatment of Diabetic Retinopathy Study (ETDRS) letters, the National Eye Institute Visual Functioning Questionnaire 25 (NEI VFQ-25) score, and foveal and retinal sensitivity in dB on microperimetry. The NEI VFQ-25 responses were converted to a score between 0 and 100. Adverse events were reported to the principal investigator and the data safety monitoring board within 24 h after the event.

### PDT procedure

In order to receive half-dose PDT, the study eye was dilated with topical 2.5% phenylephrine (phenylephrine monofree; Théa Pharma, Haarlem, the Netherlands) and 1.0% tropicamide (tropicamide monofree; Théa Pharma). Afterwards, patients received an intravenous infusion of 3 mg/m^2^ (half-dose) verteporfin (Visudyne®; Novartis, Basel, Switzerland) within 10 min. The study eye was anesthetized with oxybuprocaine 0.4% (oxybuprocaine monofree; Théa Pharma) and a PDT magnification lens was positioned on the eye. The area to be treated was determined based on hyperfluorescent area(s) on mid-phase (10 min) indocyanine green angiography (ICGA), which corresponds to macular SRF on OCT and hyperfluorescent leakage points on the mid-phase (3 min) fluorescein angiography. Laser spot size was based on the diameter of the hyperfluorescent area(s) on ICGA, plus an additional 1 mm. Subsequently, half-dose PDT was performed in the area to be treated with a fluency of 50 J/cm^2^, wavelength of 689 nm, and treatment duration of 83 s [23].

### Oral eplerenone

Eplerenone treatment consisted of 25 mg/day of oral eplerenone (Inspra®, Pfizer, Capelle aan de IJssel, the Netherlands) for 1 week, during which the serum potassium treatment was assessed. After 1 week, the dose was adjusted depending on the potassium level (the eplerenone dose was increased to 50 mg/day if the serum potassium level was < 5.0 mEq/L; the eplerenone dose remained at 25 mg/day if the serum potassium level was 5.0–5.4 mEq/L; the eplerenone dose was decreased to 25 mg every 2 days if the serum potassium level was 5.5–5.9 mEq/L; finally, the eplerenone treatment was terminated if the serum potassium level was ≥ 6.0 mEq/L). The eplerenone dose was adjusted again depending on the potassium level that was measured at 1, 2, and 3 months after the start of treatment [23]. Eplerenone is not approved by the US Food and Drug Administration for the treatment of cCSC.

### Statistics

Statistical analyses were performed using both SPSS statistics (version 25.0; IBM Corp, Armonk, New York, USA) and R (version 4.0.1; R Foundation for Statistical Computing, Vienna, Austria). A mixed effects logistics regression model was used to analyze the primary endpoint (binary longitudinal outcome of SRF on OCT), by using the function mixed_model(.) from the R package GLMMadaptive. The continuous longitudinal endpoints such as BCVA, foveal and retinal sensitivity on microperimetry, and NEI VFQ-25 score were analyzed by using a linear mixed effects mixed model. Mixed effects models have been used for the analysis to take into account the within subjects correlations induced/caused by the repeated evaluations in time. In addition, the mixed effects models give valid results under the Missing At Random assumption for the missing evaluation visits. Patients were analyzed in the subgroup that they were originally randomized to, regardless of what (crossover-) treatment they received (intention-to-treat analysis). The groups were not analyzed in 4 groups, taking crossover treatment into account, since crossover treatment was not given at random, and therefore an analysis of these 4 separate groups with a mixed model would be biased.

## Results

**Fig. 1:**
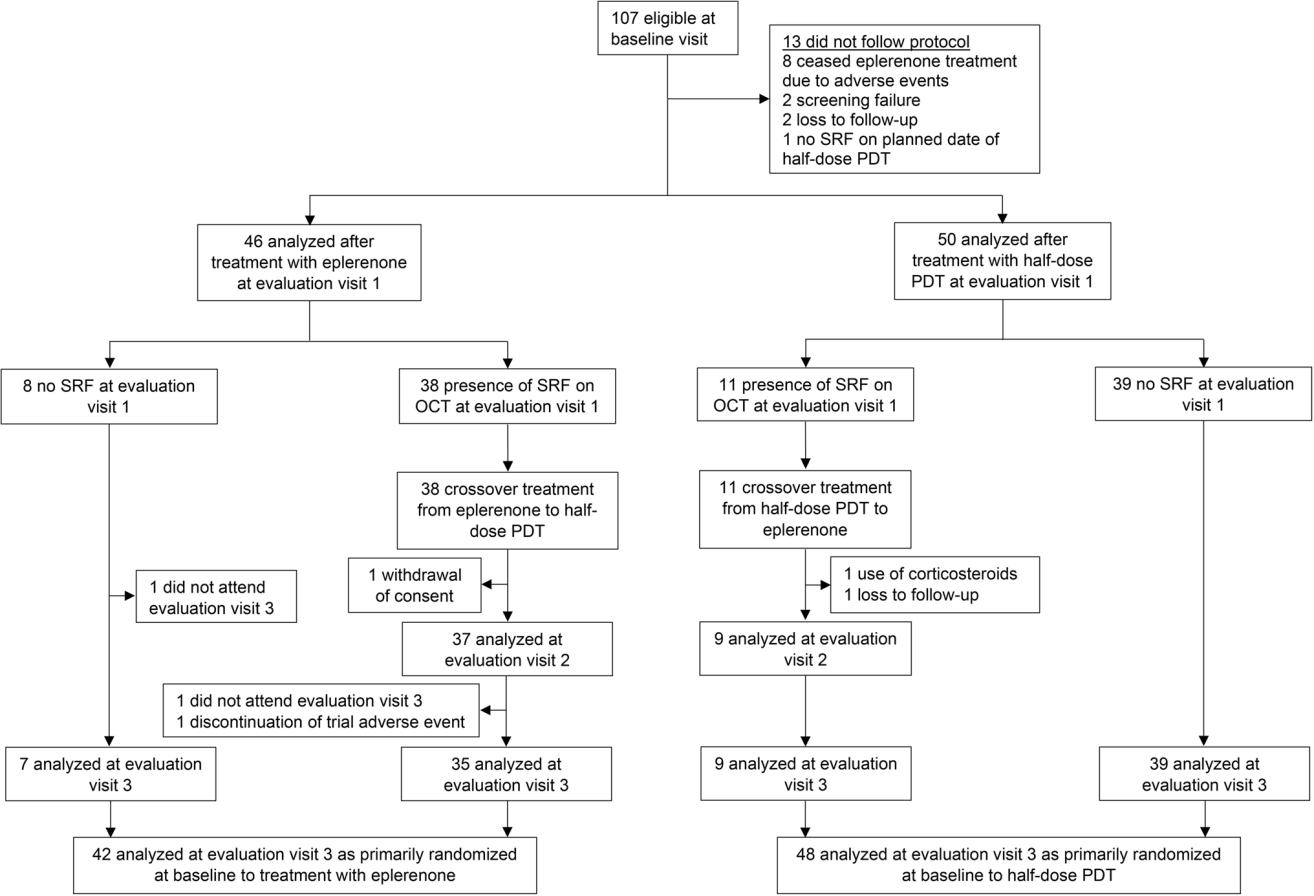
Flow-chart depicting the selection criteria for the patients in the SPECTRA trial (half-dose photodynamic therapy versus eplerenone in chronic central serous chorioretinopathy patients). OCT = optical coherence tomography; PDT = photodynamic therapy; SPECTRA = half-dose photodynamic therapy versus eplerenone in chronic central serous chorioretinopathy patients; SRF = subretinal fluid

A total of 107 cCSC patients were included in the SPECTRA trial and were analyzed at 3 months after baseline visit (evaluation visit 1). At evaluation visit 1, 11/49 (22.4%) of half-dose PDT-treated patients and 38/49 (77.6%) of the eplerenone-treated cCSC patients had persistent SRF on OCT and received crossover treatment [23, 24]. Ninety patients (84.1%) attended the visit at 12 months after baseline (evaluation visit 3); 48 of these patients were primarily randomized to half-dose PDT (of whom 9 received crossover treatment), and 42 patients to eplerenone (of whom 35 received crossover treatment). Six patients were analyzed at evaluation visit 1 but could not be analyzed at evaluation visit 3, due to no show (3), withdrawal of consent (1), use of steroids (1), or an adverse event (tiredness, dizziness, and fatigue after crossover treatment with eplerenone) (1) (Fig. 1). Baseline characteristics of the SPECTRA trial are summarized in Online Resource 2.

**Fig. 2:**
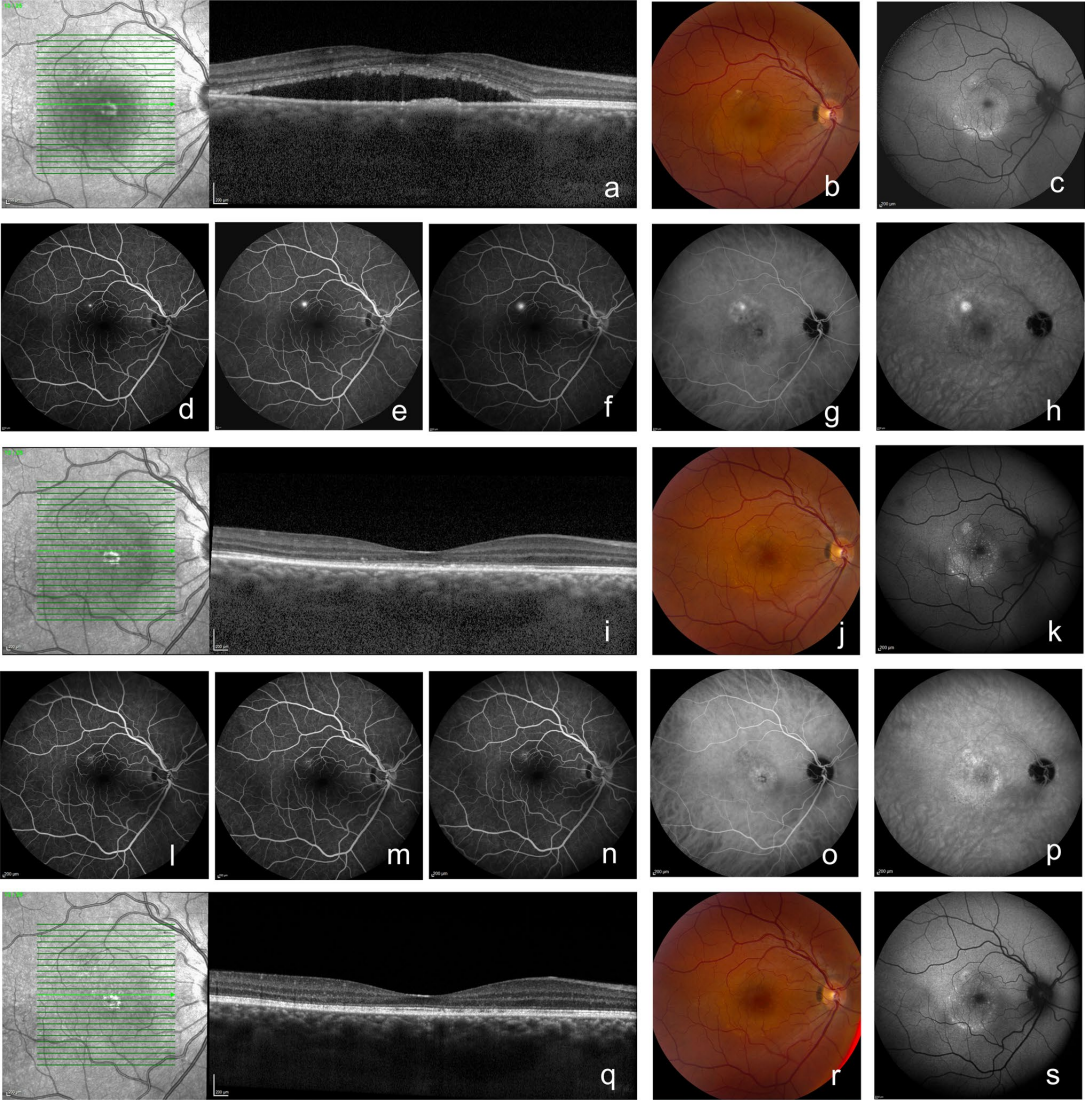
Multimodal imaging of a 34-year-old male with chronic central serous chorioretinopathy who received primary half-dose photodynamic therapy (PDT). This patient presented with subretinal fluid (SRF) on optical coherence tomography (OCT; **a**) and fundus photograph (**b**) at the baseline visit of the SPECTRA trial, before treatment with half-dose photodynamic therapy (PDT; **a–g**). At this visit, both hyper- and hypo-autofluorescent changes were visible on fundus autofluorescence imaging (FAF; **C**). An area of focal leakage was visible on fluorescein angiography (FA; **d**–**f**; early-, mid-, and late-phase, respectively), whereas hyperfluorescent abnormalities typical of central serous chorioretinopathy were seen on indocyanine green angiography (ICGA; **g–i**; early- and mid-phase, respectively). At an evaluation visit at 3 months after half-dose PDT (evaluation visit 1; **i–p**), a complete resolution of SRF on OCT had occurred (**i**), which could also be seen on the fundus photograph (**j**). FAF (**k**) did not show clear changes over time. FA (**l**, **m**, and **n**; early-, mid-, and late-phase, respectively) did not show any leakage, whereas ICGA (**o**, **p**; early and mid-phase, respectively) showed hypo- and hyperfluorescence. At 12 months after baseline visit (at evaluation visit 3; **q–s**), SRF on OCT was still absent (**q**). Both the fundus photograph (**r**) and FAF (**s**) did show any changes since evaluation visit 2. At this visit, an FA and ICGA had not been performed, in accordance with the trial protocol

**Fig. 3:**
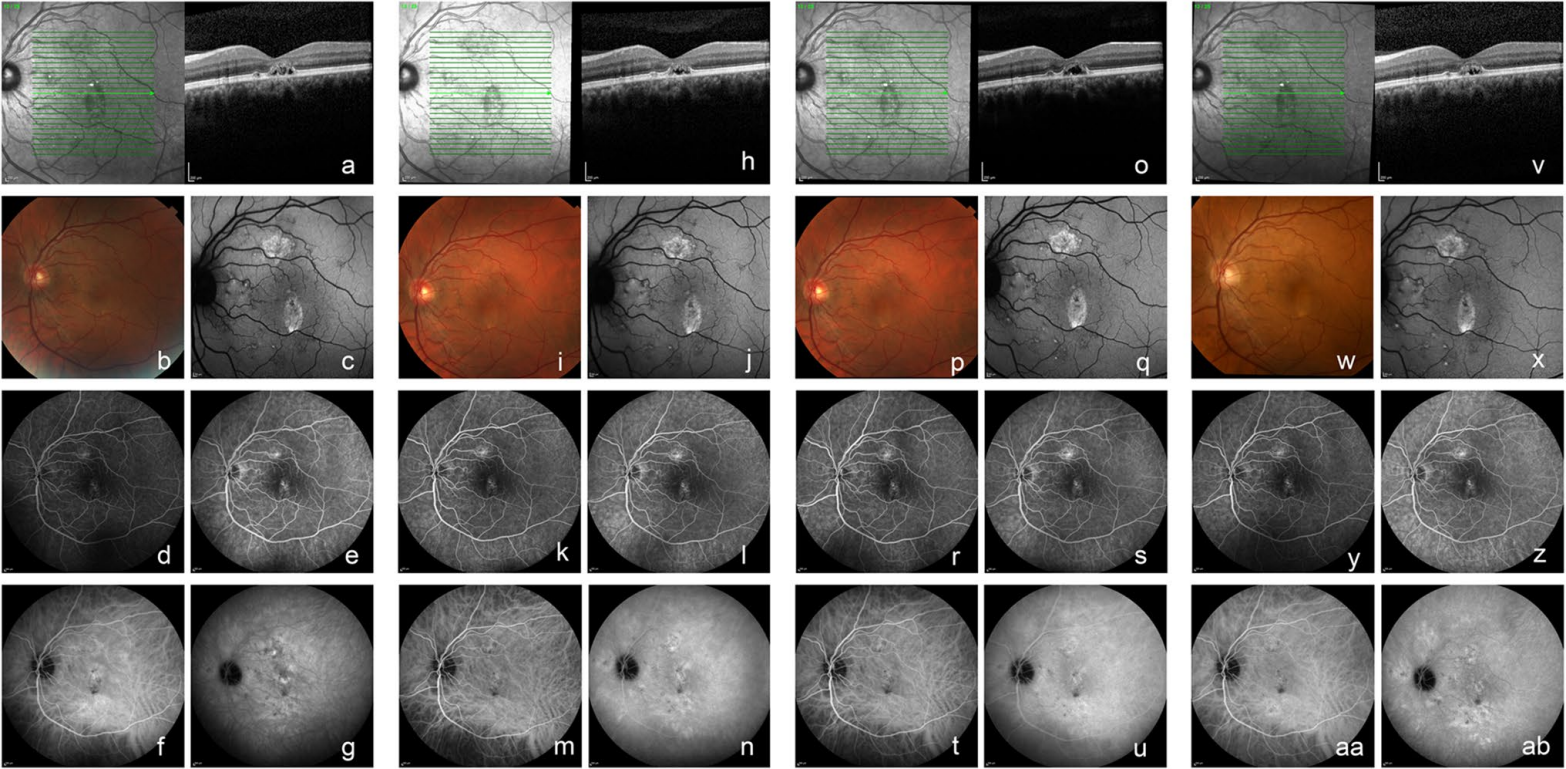
Multimodal imaging of a 42-year-old male chronic central serous chorioretinopathy patient who received crossover treatment with eplerenone, after primary failure of half-dose photodynamic therapy. At baseline visit of the SPECTRA trial (before half-dose photodynamic therapy (PDT); **a–g**), subretinal fluid (SRF) was visible on the optical coherence tomography (OCT; **a**) scan. This SRF was also visible on the fundus photograph (**b**). The fundus autofluorescence (FAF) image (**c**) showed mainly hyperautofluorescent changes. Two focal leakage points were visible on fluorescein angiography (FA; **d** and **e**; early- and late-phase, respectively), and indocyanine green angiography showed mainly hyperfluorescent abnormalities (ICGA; **f** and **g**; early- and mid-phase, respectively), with indistinct borders typical of central serous chorioretinopathy. SRF was still present on OCT (**h**), at 3 months after primary treatment with half-dose PDT (evaluation visit 1; **h–n**). The fundus photograph (**i**) and FAF (**j**) had not changed since baseline visit. The 2 focal leakage points could still be seen on FA (**k** and **l**; early and late-phase, respectively), and hyperfluorescent changes on ICGA (**m** and **n**; early- and mid-phase, respectively) had also not disappeared. The OCT scan at 3 months after crossover treatment with eplerenone (at evaluation visit 2; **o**) showed persistence of SRF. The fundus photograph (**p**) and FAF (**q**) had not changed over time. The 2 focal leakage points remained present on FA (**r** and **s**; early and late-phase, respectively**)**, whereas the early and mid-phase ICGA (**t** and **u**, respectively) still mainly revealed hyperfluorescent changes. At 12 months after baseline visit (evaluation visit 3; **v–ab**), SRF was still present on OCT (**v**). The fundus photograph (**w**) and FAF (**x**) did not show any changes. The 2 focal leakage points on the early and late-phase FA were still visible (**y** and **z**, respectively), and the hyperfluorescent changes on ICGA had also remained visible (**aa** and **ab**; early- and mid-phase, respectively)

Twelve months after baseline visit, at evaluation visit 3, complete SRF resolution was seen in 43/48 (89.6%) of patients who were primarily randomized to half-dose PDT (both with or without crossover treatment after evaluation visit 1) and in 37/42 (88.1%) of the patients who were primarily randomized to eplerenone (also both with or without crossover treatment after evaluation visit 1) (Fig. 1 and Table 1). At this visit, the odds ratio of presence of SRF on OCT did not statistically differ between the 2 treatment groups to which the patients were primarily randomized to ($\mathrm{p}$ = 0.522). If the crossover treatments were taken into account, complete SRF resolution was seen in 38/39 (97.4%) of patients with a primary resolution after PDT (who therefore did not receive crossover treatment after evaluation visit 1; Fig. 2), in 30/35 (85.7%) of the crossover from eplerenone to half-dose PDT group, 7/7 (100%) of the primary resolution after eplerenone group (who also did not receive crossover treatment), and, lastly, in 5/9 (55.6%) of the crossover from PDT to eplerenone group (Fig. 3). Of note, 2 patients in the crossover from PDT to eplerenone group and 3 patients from the crossover from eplerenone to PDT group received anti-vascular endothelial growth factor receptor injections due to persistence of SRF and a suspected choroidal neovascularization on multimodal imaging. A total of 7 patients had no SRF on OCT at evaluation visit 1 or 2, but had a recurrence of SRF on OCT at evaluation visit 3 (of whom 5 had received crossover treatment with half-dose PDT, 1 had received crossover treatment with eplerenone, and 1 patient had only been primarily treated with half-dose PDT). In 7/12 patients who had persistent SRF at evaluation visit 2, despite the fact that they had received crossover treatment, this SRF had disappeared at evaluation visit 3 (3 crossover to half-dose PDT and 4 crossover to eplerenone).

**Table 1:** Treatment effect of primary treatment with either half-dose photodynamic therapy or eplerenone on primary and secondary outcome measures in chronic central serous chorioretinopathy patients

Variable	Visit	Primarily randomized to half-dose PDT	Primarily randomized to eplerenone	p-value
Absence of SRF on OCT	Evaluation visit 1	*39/50 (78.0%)*	*8/46 (17.4%)*	
	Evaluation visit 3	43/48 (89.6%; p < 0.001)	37/42 (88.1%; p < 0.001)	0.522
Odds ratio of presence of SRF on OCT at 12 months compared to baseline	Evaluation visit 3	0.00104 (p < 0.001)	0.00159 (p < 0.001)	0.522
BCVA (ETDRS letters)	Baseline visit	78.0 (n = 53; SD = 13.1)	80.5 (n = 55; SD = 7.9)	
	Evaluation visit 3	85.9 (n = 48; SD = 11.5)	83.98 (n = 42; SD = 11.1)	
	Difference between baseline visit and evaluation visit 3	+ 7.8 (n = 48; SD = 9.8; p < 0.001)	+ 3.5 (n = 42; SD = 9.4; p = 0.018)	**0.030**
Foveal sensitivity on microperimetry (dB)	Baseline visit	20.1 (n = 49; SD = 4.6)	20.0 (n = 50; SD = 4.7)	
	Evaluation visit 3	25.5 (n = 46; SD = 5.5)	24.9 (n = 40; SD = 5.0)	
	Difference between baseline visit and evaluation visit 3	+ 5.5 (n = 42; SD = 5.2; p < 0.001)	+ 4.9 (n = 39; SD = 4.1; p < 0.001)	0.622
Retinal sensitivity on microperimetry (dB)	Baseline visit	22.4 (n = 49; SD = 4.3)	22.5 (n = 50; SD = 4.1)	
	Evaluation visit 3	26.8 (n = 46; SD = 4.0)	25.8 (n = 40; SD = 3.6)	
	Difference between baseline visit and evaluation visit 3	+ 4.5 (n = 42; SD = 3.5; p < 0.001)	+ 3.3 (n = 39; SD = 3.0; p < 0.001)	0.107
NEI VFQ-25 score	Baseline visit	81.7 (n = 53; SD = 11.3)	79.5 (n = 54; SD = 13.1)	
	Evaluation visit 3	88.6 (n = 48; SD = 9.1)	87.6 (n = 42; SD = 9.8)	
	Difference between baseline visit and evaluation visit 3	+ 7.1 (n = 48; SD = 10.4; p < 0.001)	+ 8.1 (n = 42; SD = 8.9; p < 0.001)	0.600

*BCVA*, best-corrected visual acuity; *dB*, decibel; *ETDRS*, Early Treatment of Diabetic Retinopathy Study; *HSML*, high-density subthreshold micropulse laser; *NEI VFQ-25*, National Eye Institute Visual Functioning Questionnaire 25; *OCT*, optical coherence tomography; *PDT*, photodynamic therapy; *SD*, standard deviation; *SRF*, subretinal fluid

Functional parameters were analyzed at evaluation visit 3 and compared to baseline visit and were also analyzed within the treatment groups patients were randomized to at baseline visit, without taking crossover treatments into account (Table 1). The BCVA increased with 7.8 ETDRS letters within the group primarily randomized to half-dose PDT (*p* < 0.001) and with 3.5 ETDRS letters in the group primarily randomized to eplerenone treatment (*p* = 0.018), which was a significant difference between the 2 groups (*p* = 0.030). The foveal sensitivity on microperimetry significantly increased in both the primary half-dose PDT group (+ 5.5 dB; *p* < 0.001) and the eplerenone group (+ 4.9 dB; *p *< 0.001), but there were no differences in the changes between the 2 groups (*p* = 0.622). The retinal sensitivity also increased significantly within both groups (+ 4.5 dB; < 0.001 and + 3.3; < 0.001, respectively), again without a difference between the groups (*p* = 0.107). Lastly, there were no statistical differences in change of NEI VFQ-25 score between baseline visit and evaluation visit 3 between the 2 groups (*p* = 0.600), although the increase in NEI VFQ-25 score increased significantly in both groups (+ 7.1 points; *p* < 0.001 in the half-dose PDT group vs + 8.1 points; *p* < 0.001 in the eplerenone treatment group).

Out of the 46 patients who analyzed after treatment with eplerenone at evaluation visit 1, 42 (91.3%) received an eplerenone dose of 50 mg/day after the first week of treatment initiation, while 4/46 (8.7%) received a dose of 25 mg/day. All of the 9 patients who were analyzed at evaluation visit 2 after crossover treatment with eplerenone after failure or primary half-dose PDT received 50 mg/day of eplerenone after the first week of initiation of the treatment. The adverse events that occurred between baseline and evaluation visit 1 have been described in a previous publication and have also been included in Online Resource 3 [25]. In the group of patients primarily randomized to half-dose PDT, 10/50 (20.0%) patients had an adverse event between evaluation visit 1 and 3. In this group, only 1 adverse event was possibly treatment-related (paresthesia of hand or leg after crossover treatment with eplerenone). In the group that was primarily randomized to eplerenone, adverse events were present in 10/46 (21.7%) patients between evaluation visit 1 and 3, with 2 events being possibly treatment-related (visual complaints in both eyes after crossover treatment with half-dose PDT and an itchy eye lid after primary eplerenone treatment). None of the adverse events were definitely treatment-related. There were no serious adverse events.

## Discussion

To date, no large randomized controlled trial has been reported on the long-term results of oral eplerenone in comparison to half-dose PDT in cCSC. In the current trial, we evaluated the long-term outcomes after treatment with half-dose PDT, eplerenone, and, in some cases (especially in primary eplerenone-treated patients), with crossover treatment. This study included a wide variety of cCSC patients, from focal to diffuse variants. We show that almost all patients treated with half-dose PDT still have a complete SRF resolution at 12 months after baseline, including the patients who received half-dose PDT as crossover treatment. Remarkably, BCVA also increases significantly more in the patients originally randomized to half-dose PDT compared to patients who were primarily randomized to eplerenone treatment, despite the crossover treatment with half-dose PDT which most of these patients received because of persistent SRF on OCT.

The odds ratio of complete SRF resolution on OCT at 12 months after baseline visit (evaluation visit 3) did not differ statistically between the 2 treatment groups to which patients were primarily randomized to. However, it should be noted that due to the intention-to-treat design of the trial, 82.6% of the patients who were primarily randomized to the eplerenone group received crossover treatment with half-dose PDT due to persistence of SRF on OCT at evaluation visit 1, whereas only 22.0% of patients from the primary half-dose PDT group required crossover treatment with eplerenone (Fig. 1). As a result, most patients analyzed at evaluation visit 3 had received treatment with half-dose PDT (92.2%), either as primary or as crossover treatment.

The results of the current study suggest that cCSC patients benefit most from treatment with half-dose PDT, even after previous unsuccessful treatment with eplerenone for 3 months. This is in line with the available evidence from randomized controlled trials (SPECTRA, SPECS, PLACE, REPLACE, and VICI) which have shown that half-dose PDT is superior to high-density subthreshold micropulse laser treatment and oral eplerenone in achieving a complete SRF resolution on OCT in the short term [19–24]. SRF in cCSC has been associated with reduced long-term BCVA outcomes and decreased quality of life [18]. In the current study, we have also found a significantly larger increase in BCVA in ETDRS letters in cCSC patients primarily randomized to half-dose PDT compared to eplerenone, despite the fact that a large portion of the eplerenone group received crossover treatment with half-dose PDT. This difference between the 2 groups could be explained by the fact that, on average, SRF was present for a longer period in patients who were primarily randomized to eplerenone compared to the patients randomized to half-dose PDT. Previous studies have also suggested that a prolonged presence of SRF can irreversibly damage the retina [16, 26]. Both the placebo group and the eplerenone group of the VICI study showed an increase of BCVA of only 4 ETDRS letters at 12 months after baseline visit, which is lower than the 7.8 ETDRS letters found in the primary half-dose PDT group of our study, but similar to the findings in our primary eplerenone group [22]. The remaining functional parameters (NEI VFQ-25 score, and retinal and foveal sensitivity on microperimetry) significantly increased within both groups (*p* < 0.001), but there were no significant differences between the groups. Therefore, it appears important not to postpone PDT and to avoid attempting treatment modalities with lower levels of evidence if PDT is available [16, 18, 26]. The superiority of half-dose PDT over other available treatments may be explained by the fact that PDT presumably treats the choroid, which is thickened and leaky in cCSC patients, causing choroidal remodeling [4, 27–29]. This process may restore the balance between the damaged retinal pigment epithelium outer blood-retina barrier and the choroid, with subsequent resolution of SRF [30]. Lastly, it is important to note that treatment-related adverse events were more common in cCSC patients in the SPECTRA trial who received eplerenone compared to half-dose PDT [23].

This study has some limitations. Firstly, this study was powered for the 2 treatment groups the patients were randomized to at baseline. After crossover treatment, statistical analysis with a mixed model would be biased, as the 4 groups were not divided at random. Therefore, we used an intention-to-treat model for the statistical analysis of this study. Moreover, these groups were largely disproportionate after evaluation visit 1, due to the much higher success rate of half-dose PDT compared to oral eplerenone treatment. Another bias is the fact that a minority of cCSC patients did not attend evaluation visit 3.

In conclusion, most cCSC patients treated with half-dose PDT, either as primary treatment or crossover treatment after initial unsuccessful eplerenone treatment, achieve a complete resolution of SRF on OCT without recurrences at 12 months after baseline. BCVA improved significantly more in cCSC patients who received half-dose PDT as their first treatment compared to patients who first received unsuccessful oral eplerenone treatment followed by crossover half-dose PDT, although retinal sensitivity, microperimetry, and NEI VFQ-25 score did not differ between these groups. The results of our study add to the growing evidence that relatively early half-dose PDT is the first treatment of choice for cCSC, both in terms of anatomical and functional outcomes.

## Supplementary Information

Below is the link to the electronic supplementary material.

Supplementary file1 (PDF 14 KB)

Supplementary file2 (PDF 31 KB)

Supplementary file3 (PDF 77 KB)
