# Supplementary figures and images for: Long-term follow-up of chronic central serous chorioretinopathy patients after primary treatment of oral eplerenone or half-dose photodynamic therapy and crossover treatment: SPECTRA trial report No. 3
Source: Graefes Arch Clin Exp Ophthalmol. 2022 Oct 7;261(3):659–68. doi: 10.1007/s00417-022-05836-x (PMC9988736; doi:10.1007/s00417-022-05836-x)

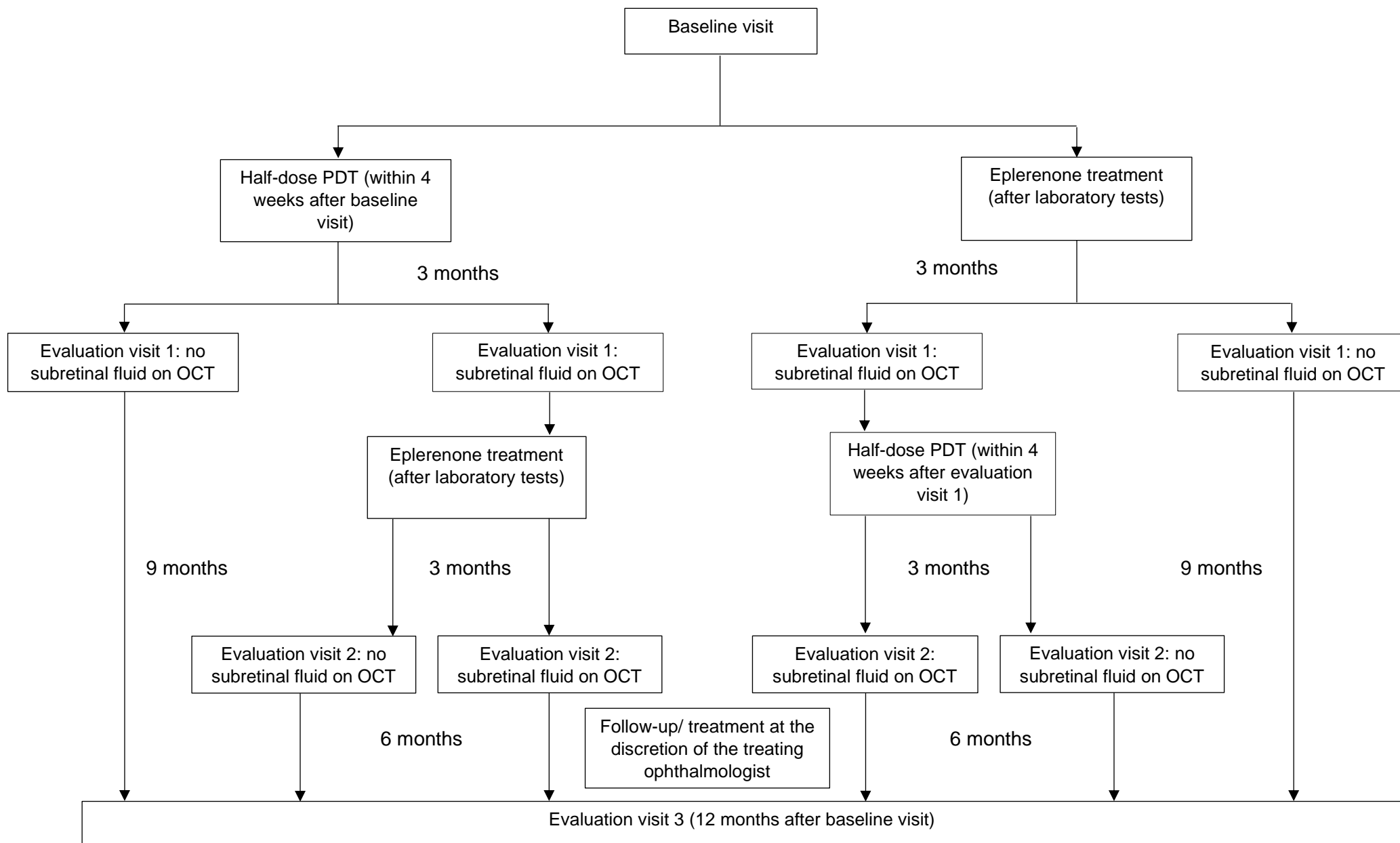

Supplement: Supplementary file 1 — Supplementary file1 (PDF 14 KB) [file 417_2022_5836_MOESM1_ESM.pdf]
