# Supplementary material for: Long-term follow-up of chronic central serous chorioretinopathy patients after primary treatment of oral eplerenone or half-dose photodynamic therapy and crossover treatment: SPECTRA trial report No. 3
Source: Graefes Arch Clin Exp Ophthalmol. 2022 Oct 7;261(3):659–68. doi: 10.1007/s00417-022-05836-x (PMC9988736; doi:10.1007/s00417-022-05836-x)
Supplement: Supplementary file 2 — Supplementary file2 (PDF 31 KB) [file 417_2022_5836_MOESM2_ESM.pdf]

	**Half-dose photodynamic therapy group (n=53)**	**Eplerenone group (n=54)**	**Total (n=107)**
Mean age (years)	44.5 ± 10.0	47.5 ± 9.6	46.0 ± 9.9
Male gender	51 (96%)	49 (91%)	100 (94%)
Mean best-corrected visual acuity (ETDRS letters)	78.0 ± 13.1	80.5 ± 7.9	79.3 ± 10.8
Mean retinal sensitivity on microperimetry (dB)	22.7 ± 4.3	22.5 ± 4.1	22.6 ± 4.2
Mean NEI-VFQ-25 composite score (points)	81.7 ± 11.3	79.4 ± 13.1	80.5 ± 12.2
