# Supplementary material for: Long-term follow-up of chronic central serous chorioretinopathy patients after primary treatment of oral eplerenone or half-dose photodynamic therapy and crossover treatment: SPECTRA trial report No. 3
Source: Graefes Arch Clin Exp Ophthalmol. 2022 Oct 7;261(3):659–68. doi: 10.1007/s00417-022-05836-x (PMC9988736; doi:10.1007/s00417-022-05836-x)
Supplement: Supplementary file 3 — Supplementary file3 (PDF 77 KB) [file 417_2022_5836_MOESM3_ESM.pdf]

**Baseline – evaluation visit 3**				
	**Primarily randomized to half-dose PDT group (n=53)**		**Primarily randomized to eplerenone (n=54)**	
	Number of patients (%)	Number of events	Number of patients (%)	Number of events
All adverse events	13 (24.5%)	16	26 (48.1%)	33
Serious adverse events	0	0	0	0
Number of patients reporting the following symptoms throughout the trial				
Stomach complaints after taking medication (not eplerenone)	1 (not treatment-related)			
Nausea after taking medication (varenicline)			1 (not treatment-related)	
Nausea			1 (possibly treatment-related)	
Stomach complaints			2 (possibly treatment-related)	
Cataract extraction			1 (not treatment-related)	
Cataract (not treated)			1 (not treatment-related)	
Itchy eye lid			1 (possibly treatment-related)	
Headache			3 (possibly treatment-related)	
Visual complaints in both eyes			1 (possibly treatment-related)	
Vertigo	1 (not treatment-related)			
Dizziness			3 (possibly treatment-related)	
Skin rash	1 (not treatment-related)		2 (not treatment-related)	
Fatigue, poor quality of sleep	1 (not treatment-related)		1 (not treatment-related)	
General malaise			1 (possibly treatment-related)	
Lower back pain	1 (not treatment-related)			
Cornea erosion	1 (not treatment-related)			
Discovery of benign kidney cysts			1 (not treatment-related)	
Removal of benign warts			1 (not treatment-related)	
Paresthesia hand or leg	1 (possibly treatment-related)		2 (possibly treatment-related)	
Eczema	1 (not treatment-related)		1 (not treatment-related)	
Vasovagal reaction during fluorescein angiography			2 (possibly treatment-related)	
High glucose levels during routine check	1 (not treatment-related)			
Tonic-clonic seizure	1 (not treatment-related)			
Head injury	1 (not treatment-related)			
Folliculitis	1 (not treatment-related)			
Dental pain	1 (not treatment-related)			
Rib contusion	1 (not treatment-related)			
Sprained ankle			1 (possibly treatment-related)	
Rhinitis			1 (not treatment-related)	
Diarrhea			1 (possibly treatment-related)	
Nipple tenderness			1 (possibly treatment-related)	
Heart palpitations			1 (possibly treatment-related)	
Neovascularization in study eye	2 (not treatment-related)		3 (not treatment-related)	
